# Supplementary material for: DFT Study of Adsorption Behavior of Nitro Species on Carbon-Doped Boron Nitride Nanoribbons for Toxic Gas Sensing
Source: Nanomaterials (Basel). 2023 Apr 19;13(8):1410. doi: 10.3390/nano13081410 (PMC10143892; doi:10.3390/nano13081410)
Supplement: Supplementary file 1 [file nanomaterials-13-01410-s001.zip › nanomaterials-2341342-supplementary.pdf]

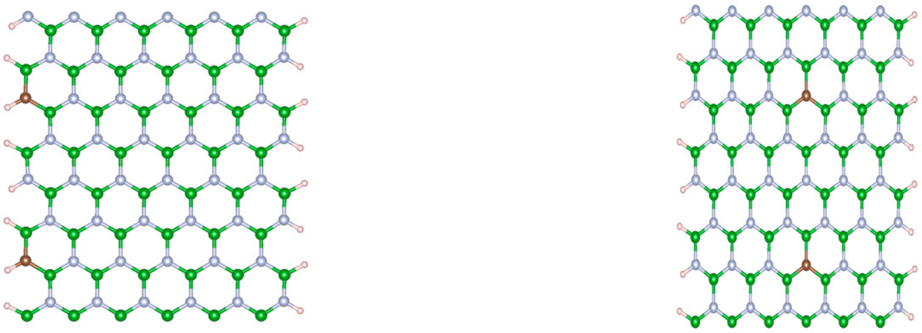

(**a**) (**b**)

Figure S1. Schematic representation of a M-BNNR with 4-member rings in the periodic direction: (**a**) E_M-BNNR and (**b**) C_M-BNNR.

Optimized coordinates for pristine BNNR, 12 x 2

N	0.985679	7.807322	5.000000
B	2.198260	7.114852	5.000000
N	3.423148	7.814341	5.000000
B	3.423509	9.239210	5.000000
N	2.198569	9.937736	5.000000
B	0.986549	9.243822	5.000000
B	4.644809	7.117476	5.000000
N	4.645104	5.676537	5.000000
B	3.423518	4.979245	5.000000
N	2.198464	5.677745	5.000000
N	5.857539	7.810951	5.000000
B	7.073721	7.117484	5.000000
N	7.073848	5.687485	5.000000
B	5.857965	4.983118	5.000000
N	8.288149	7.806510	5.000000
B	9.503527	7.111809	5.000000
N	9.504644	5.667449	5.000000
B	8.289373	4.972364	5.000000
N	10.720164	7.806357	5.000000
B	11.949442	7.107652	5.000000
N	11.949864	5.675908	5.000000
B	10.721711	4.973059	5.000000
N	13.161531	7.803093	5.000000
B	13.163227	9.244282	5.000000
N	11.949749	9.935829	5.000000
B	10.721297	9.233042	5.000000
N	9.504478	9.927523	5.000000
B	9.504099	11.371975	5.000000
N	10.720944	12.066503	5.000000
B	11.949881	11.367628	5.000000
N	14.383762	9.942282	5.000000
B	14.383288	11.367161	5.000000
N	13.161845	12.063191	5.000000

B	8.289110	9.232405	5.000000
N	7.086427	9.943798	5.000000
B	7.086328	11.370295	5.000000
N	8.288730	12.066617	5.000000
B	13.163311	4.984314	5.000000
N	14.383723	5.682323	5.000000
B	14.382955	7.107149	5.000000
B	5.857958	9.243038	5.000000
N	4.645090	9.936511	5.000000
B	4.644826	11.377431	5.000000
N	5.857536	12.070940	5.000000
B	2.198367	11.374793	5.000000
N	3.423183	12.074311	5.000000
B	0.986469	4.983891	5.000000
N	0.985837	12.067333	5.000000
H	15.329713	5.142857	5.000000
H	15.328364	7.647634	5.000000
H	15.328656	11.907716	5.000000
H	0.161334	5.694575	5.000000
H	0.045159	7.258374	5.000000
H	0.161394	9.954481	5.000000
H	0.161418	11.355819	5.000000
H	15.324617	9.393909	5.000000

Optimized coordinates for E_M-BNNR, 12 x 2

H	20.441330	0.863552	5.000000
H	20.666704	3.338318	5.000000
H	20.666977	7.707554	5.000000
H	4.721060	1.128165	5.000000
H	4.753009	3.042279	5.000000
H	4.717142	5.695369	5.000000
H	4.975795	7.403604	5.000000
H	20.440884	5.232770	5.000000
C	5.712397	3.557647	5.000000
N	7.026066	5.724326	5.000000
N	5.844355	7.929697	5.000000
N	7.044511	1.327978	5.000000
N	9.545887	5.699317	5.000000
N	8.315241	7.895314	5.000000
N	9.558711	1.338223	5.000000
N	12.068054	1.335342	5.000000
N	14.578456	1.336110	5.000000
N	17.088339	1.337705	5.000000
N	19.562565	1.371161	5.000000
N	18.352470	3.522330	5.000000
N	19.562466	5.740935	5.000000
N	18.352993	7.892292	5.000000
N	15.835560	7.891638	5.000000

N	13.323611	7.890264	5.000000
N	10.813334	7.888776	5.000000
N	10.810425	3.520285	5.000000
N	12.064280	5.704358	5.000000
N	14.577353	5.705993	5.000000
N	17.087910	5.707704	5.000000
N	13.322671	3.520877	5.000000
N	15.835262	3.521744	5.000000
N	8.291995	3.515263	5.000000
B	5.742583	5.060599	5.000000
B	7.060149	7.177559	5.000000
B	7.026217	2.780572	5.000000
B	5.790839	0.596126	5.000000
B	8.286075	4.969545	5.000000
B	9.560832	7.157945	5.000000
B	8.307312	0.605171	5.000000
B	10.813352	0.606414	5.000000
B	13.323753	0.606281	5.000000
B	15.834053	0.606918	5.000000
B	18.343945	0.607729	5.000000
B	19.596584	2.785650	5.000000
B	18.343637	4.977801	5.000000
B	19.596966	7.155382	5.000000
B	17.093334	7.162684	5.000000
B	14.580769	7.161287	5.000000
B	12.070251	7.159215	5.000000
B	9.551381	2.794535	5.000000
B	10.807987	4.973149	5.000000
B	13.321663	4.975832	5.000000
B	15.833324	4.976801	5.000000
B	12.067060	2.791247	5.000000
B	14.579971	2.791917	5.000000
B	17.093010	2.792726	5.000000

Optimized coordinates for C_M-BNNR, 12 x 2

H	20.154916	0.964900	5.000000
H	20.395007	3.439412	5.000000
H	20.376043	7.816961	5.000000
H	20.149124	5.341263	5.000000
H	4.377785	1.259121	5.000000
H	4.595601	3.164114	5.000000
H	4.381659	5.639717	5.000000
H	4.609874	7.522076	5.000000
C	13.008688	3.640117	5.000000
N	5.476446	3.668228	5.000000
N	6.695943	5.818125	5.000000
N	5.486328	8.033894	5.000000
N	6.690967	1.447221	5.000000

N	9.218603	5.817145	5.000000
N	7.961830	8.003595	5.000000
N	9.209694	1.450165	5.000000
N	11.723803	1.434685	5.000000
N	14.291681	1.434475	5.000000
N	16.803885	1.449943	5.000000
N	19.279128	1.477720	5.000000
N	18.080010	3.632590	5.000000
N	19.271922	5.851667	5.000000
N	18.061448	8.001420	5.000000
N	15.546300	8.007016	5.000000
N	13.008098	8.019036	5.000000
N	10.470505	8.007574	5.000000
N	10.451564	3.632167	5.000000
N	11.735053	5.851980	5.000000
N	14.279153	5.851740	5.000000
N	16.793536	5.816655	5.000000
N	15.566846	3.631679	5.000000
N	7.947073	3.634706	5.000000
B	16.799490	7.273011	5.000000
B	5.450270	5.083270	5.000000
B	6.706439	7.273256	5.000000
B	6.694794	2.903709	5.000000
B	5.449237	0.708687	5.000000
B	7.955477	5.088066	5.000000
B	9.219186	7.273949	5.000000
B	7.953919	0.719307	5.000000
B	10.466519	0.715612	5.000000
B	13.007945	0.731016	5.000000
B	15.548247	0.714813	5.000000
B	18.057270	0.717323	5.000000
B	19.321074	2.892750	5.000000
B	18.054573	5.086813	5.000000
B	19.305646	7.265073	5.000000
B	14.281403	7.296047	5.000000
B	11.735498	7.296291	5.000000
B	9.201746	2.907258	5.000000
B	10.479825	5.093498	5.000000
B	13.007234	5.147452	5.000000
B	15.533482	5.092566	5.000000
B	11.699445	2.886318	5.000000
B	14.319225	2.886144	5.000000
B	16.819304	2.906661	5.000000

Cell latices for the BNNRs

25.3779602051	0.0000000000	0.0000000000
0.0000000000	8.7400000000	0.0000000000
0.0000000000	0.0000000000	10.0000000000

Optimized coordinates for NH4+

N	0.000000	0.000000	0.000000
H	0.000000	0.000000	1.008000
H	0.950353	0.000000	-0.335996
H	-0.475176	-0.823029	-0.336000
H	-0.475176	0.823029	-0.336000

Optimized coordinates for NH3

N	0.000000	0.000000	0.000000
H	0.000000	0.000000	1.008000
H	0.950353	0.000000	-0.335996
H	-0.475176	-0.823029	-0.336000

Optimized coordinates for NO2

N	0.000000	0.000000	0.000000
O	0.000000	0.000000	1.400000
O	1.319934	0.000000	-0.466662
